# Mr. Guthrie on Gunshot Wounds

**Published:** 1827-10

**Authors:** 


### VIII.

*A Treatise on Gun-shot Wounds, on Inflammation, Erysipelas, and Mortification, on Injuries of the Nerves, and on Wounds of the Extremities, &c. &c. &c.* By G. J. GUTHRIE, F.R.S. Surgeon to the Westminster Hospital, &c. Octavo, pp. 559, with five plates. London, 1827.

THERE was a time, and that not very long ago, when the mighty DONS of our schools and hospitals looked down with sovereign contempt on the practice of the medical officers of our fleets and armies—and deigned not to read any observations which the latter ventured to publish. Sometimes, indeed, their high mightinesses condescended to repair to our naval and military hospitals, *after battle*, in order to pick up broken bones, and such little things as might serve to make their pupils stare in the succeeding winter, or even furnish matter for sarcasm on the ignorance of naval and military surgeons. On one occasion, when the trump of war sounded so close to the chalky cliffs, that it could almost be heard there, the field of battle was covered with patriot surgeons, who gathered more laurels there, than those who toiled through the bloody fight. The schools ring to this hour with the prowess of the teachers who performed such unheard-of exploits, and ventured their precious persons in the tainted atmosphere of Waterloo, some days after the battle.

But the times are altered. The medical officers of armies and fleets have planted themselves in the same field with their *civil* brethren, and the tables are turned. The former class have pretty well proved, that talent and observation are not the exclusive property of this or that school or hospital—and that those who have toiled on the field of battle can compete in private life, with those who looked with scorn on the experience gained in the cock-pit or on the pack-saddle. It is not our intention, however, to rake up old grievances, though we could retaliate with terrible effect on our traducers. At the same time, we shall not fail to enforce the claims of our military and naval brethren to such improvements as have originated with them. On this account, we shall review the principal subjects contained in the very enlarged and improved edition of Mr. Guthrie's work now before us, in the next and the succeeding Number of this Journal. At present we can do no more than announce the new edition, and introduce the preface attached to it:

"When I printed the first edition of this work in 1815, I stated, that it contained 'many opinions in opposition to those received in common by the profession, and even now taught.' I also said, that in publishing them I was desirous of making known 'what had been the practice of the surgeons of the British army during the Peninsular war, and to preserve for them the credit of improvements, which they alone have introduced into the science and art of surgery, and particularly in the operative part, in which they have been eminently successful.' In referring to my professional brethren that credit which was their due, I by no means wished to exonerate myself from any blame that might be attached to the practice recommended, for I was aware that some of these opinions were not common to the whole, and for these in particular, as well as for every one of them, I held myself responsible. I was contented to allow them to find their way as unobtrusively as possible into the world, satisfied they would stand the test of investigation, and be ultimately adopted as principles. In this I was not mistaken; they have not only been generally adopted, but pirated by some persons, and even advanced as something new by others, many years after I had published them. In order to put a stop to such proceedings, I shall now enumerate those points in which surgery is indebted for its improvement to the medical department of the army and the practice of the Peninsular war; and in so doing I trust I shall redeem the pledge given to the medical officers of the different branches of the public service, in the introductory lecture to my first course of lectures on surgery in 1816, that I would always defend and maintain their right to the improvements they had suggested or made against all encroachment.

"Previously to the termination of the war in 1815, and the appearance of the first edition of this work, the opinions of Mr. Hunter on the powers and capabilities of the human constitution were universally received. As general principles they did little mischief; but when they came to be acted upon, the results were not found to coincide with the principles from which they were deduced. When an injury had occurred to a person in health, rendering the loss of a limb necessary, he recommended that an operation should not be performed until after suppuration had been established, a period probably of six weeks, which, even if the patient survived, was often found to be too late to be serviceable. From the failure of this practice, the contrary one of immediate amputation became gradually more general during the war, and, at its close, I not only advocated and established the propriety of it, but examined the reasoning on which Mr. Hunter's opinions were founded, and I trust have proved it to be defective. That it was so ought indeed to have been presumed, when the facts were found to be opposed to the reasons.

"It was not, however, on the single point of amputation that this reasoning led into error, it embraced the whole subject of inflammation and its consequences, which I believe can only be consistently viewed on the principles regarding the human constitution which I have advanced. The variations in the nature and appearances of erysipelas may through them be more easily comprehended, and the treatment of mortification more scientifically undertaken. The Baron Larrey had shown, that, in gangrene from wounds, amputation might occasionally be resorted to with success during its progress, in opposition to the received opinions of the schools; but he did not explain that this was entirely dependent on the circumstance of its being local. The division I have made into constitutional and local mortification, and the practice I have indicated to be followed in the different species of gangrene, from whatever causes they may have originated, as dependent on this distinction, are improvements which many are inclined to adopt, without being aware to whom they are indebted for them. There is still, however, an unaccountable slothfulness in some in neglecting all inquiry into this subject, whilst there is in others an obstinate adherence to the old practice, although invariably unsuccessful.

"The practice of the Peninsular war led, however, to another important result in surgery; it dissipated that delusion, which had so long obtained possession of the minds of surgeons of every description, 'that it was impossible to command the flow of blood through the great arteries.' I overturned at once this hypothesis, declared it to be visionary, and not only without foundation, but the reverse of fact. On the return of the medical officers of the army to London in 1814, it was not a little amusing to them to hear teachers of surgery gravely informing their students, that amputation at the shoulder-joint was a most formidable operation, on account of the impossibility of effectually preventing the flow of blood through the arteries; and when they did notice amputation of the hip-joint, it was only to declare it a murderous operation. What is the state of things now? What has the short space of twelve years done for this branch of surgery? Why almost too much. The facility with which these operations can be performed, and the safety which attends them, has been shown, and all alarm has been banished from the minds of surgeons on these points. It is now to be feared, that they may become unmindful of the precepts I have laid down demonstrating their necessity, and recommend them to be performed when others less important might suffice.

"The practice of the Peninsular war was decisive on many other points. It overturned the application of the theory of aneurism to the treatment of wounded arteries; and my paper on wounded arteries, published in 1811, in the New Medical and Physical Journal, demonstrated the necessity which existed for performing the operation at the wounded part of the vessel, and not at a distance. It showed, what is not yet well understood by many, that in no case (and this is without any exception) should one ligature above the wound be depended upon, but that another should be applied below it.

"I have proved from official documents, that the great dread entertained of secondary hæmorrhage in Gun-shot Wounds was groundless, whilst the practice in all cases has been established on more certain principles than heretofore. These and many other minor points I do not think it necessary to notice. A careful examination of the books which existed at the commencement of the Peninsular war, and a comparison of them with the observations there made on the same subject, will show in what part the alterations and improvements have taken place; whilst the work, from its continual reference to the different periods of the war, demonstrates the fact of the particular time at which each of them was established, if it does not mark that at which they originated."

